# Comparative Biochemical and Histopathological Studies on the Efficacy of Metformin and Virgin Olive Oil against Streptozotocin-Induced Diabetes in Sprague-Dawley Rats

**DOI:** 10.1155/2018/4692197

**Published:** 2018-11-18

**Authors:** Khadijah Saeed Balamash, Huda Mohammed Alkreathy, Elham Hamed Al Gahdali, Sawsan Omer Khoja, Aftab Ahmad

**Affiliations:** ^1^Department of Biochemistry, Faculty of Science, King Abdulaziz University, Jeddah, Saudi Arabia; ^2^Department of Pharmacology, Faculty of Medicine, King Abdulaziz University, Jeddah, Saudi Arabia; ^3^Health Information Technology Department, Jeddah Community College, King Abdulaziz University, P.O. Box 80283, Jeddah 21589, Saudi Arabia

## Abstract

Treatment of diabetic patients with antioxidant, such as extra virgin olive oil (EVOO), may be beneficial in numerous debilitating complexities. This study was aimed at assessing the protective role of virgin olive oil in reducing hyperglycemia in streptozotocin- (STZ-) induced diabetic rats. Thirty-six healthy male Sprague-Dawley rats were divided into six groups (6 rats per group) including nondiabetic control (NC), diabetic control (DC), and animals treated with metformin, olive oil, and a combination of olive oil and metformin, respectively. The protective effect of olive oil was evaluated by determining the biochemical parameters (lipid profile, liver, and kidney) and by studying the histopathological alterations in pancreas, liver, and kidney tissues. The results showed a significant increase in alanine aminotransferase (ALT) and alkaline phosphatase (ALP) levels in diabetic rats. ALP levels remained significantly elevated in the diabetic rats that were treated with metformin and/or olive oil, and the highest level was noted in the group treated with olive oil (568.33 U/L). Contrarily, pretreatment with olive oil significantly decreased ALT (67.64 U/L) and ALP (226.17 U/L) levels. Histopathological data revealed that all the disorganized islets of Langerhans along with the clusters of inflammatory cells were absent in the group pretreated with the combination of virgin olive oil and metformin, which shows that prophylactic administration of this combination reduces the diabetic complications in a much better way. Therefore, pretreatment with olive oil with or without metformin is an encouraging approach for diabetes therapy with immense potential.

## 1. Introduction

The incidence of diabetes, a metabolic disorder, has soared globally in the last three decades mainly due to lifestyle modifications [1, 2]. It is estimated that by the year 2040, the number of diabetic adults worldwide would reach 642 million [3].

Diabetes mellitus (DM) is a global health issue with an ever increasing prevalence. The primary causes for the global ascendancy in DM are as follows: increase in aged population, stationary way of life, expanding patterns towards obesity, and unhealthy diet [1, 2]. The symptoms of DM consist of increased hunger, increased thirst, weight loss, polyuria, obscuring of vision, irregularities of lipoprotein metabolism, and hypertension. Severe complexities of diabetes mellitus like nonketotic hyperosmolar state or ketoacidosis might develop and may cause stupor, coma, and death [4]. The long-standing effects of diabetes mellitus involve a gradual advancement of the particular complexities of retinopathy with potential visual impairment, nephropathy that may prompt renal impairment, and/or neuropathy with danger of foot ulcers, Charcot joints, and features of autonomic dysfunction [5].

Since diabetes is known to induce an increase in free radical production that further leads to oxidative stress and damage of cell components, it is hypothesized that an antioxidant therapy could help to prevent diabetic complications [6]. Antioxidants might prevent the development of diabetes by repressing the peroxidation chain reaction [7, 8]. Numerous routine antioxidants like vitamin E and phenolic compounds are known to have hypoglycemic and/or hypolipidemic activities [9]. Though many antidiabetic medications are available, safer organic sources are preferred by diabetic patients.

Olive oil is one such natural agent that is a high source of phenols and is known to induce protective effects against many diseases [10]. The phenolic compounds of olive oil have anti-inflammatory and antioxidant properties and also have favorable impact on different physiological parameters, such as antimicrobial actions, bone validity, inflammatory markers, oxidative harm, plasma lipoproteins, and platelet and cell capacity [10–12]. Diet rich in olive oil is also known to mitigate diabetes [13, 14].

Therefore, this study is aimed at assessing the protective role of virgin olive oil in reducing hyperglycemia in STZ-induced diabetic rats by determining serum biochemical parameters and by studying the histopathological alterations of the pancreas, liver, and kidney.

## 2. Materials and Methods

### 2.1. Materials

Virgin olive oil (VOO) was obtained from Aladhara agricultural project, Al-Jouf, Kingdom of Saudi Arabia (KSA). Streptozotocin (STZ) was purchased from Sigma-Aldrich, USA. Metformin as glucophage (1000 mg tablet) was procured from a local pharmacy and was dissolved in distilled water containing 0.9% (wt/vol) sodium chloride for oral administration.

### 2.2. Animals

A total of 36 male Sprague-Dawley rats weighing 200-250 g were used for the study. The rats were housed in plastic cages at a controlled temperature of 22 ± 3°C with a 12-hour light/dark cycle for two weeks. Animals were fed with standard chow and supplied with drinking water ad libitum. The study lasted for a total of 6 weeks.

The protocol of this study was approved by the Unit of Biomedical Ethics, Faculty of Medicine, King Abdulaziz University, Jeddah, Saudi Arabia. All animal procedures and experiments were performed according to King Abdulaziz University's policy and international ethical guidelines on the care and use of laboratory animals (the National Research Council of the National Academy of Sciences, 2011).

### 2.3. Methods

#### 2.3.1. Induction of Diabetes

Streptozotocin (STZ) was dissolved in 0.05 M citrate buffer (pH 4.5; 1 mL). Diabetes was induced by giving a single intraperitoneal (IP) injection of STZ (40 mg/kg bw). [15]. After STZ injection, rats were given 10% (wt/vol) ad libitum fructose solution for 3 days. Blood glucose level was measured after 3 days. Animals with blood glucose level above 250 mg/dL were considered diabetic and were used in the experiment. Rats were divided into six groups: group 1 comprised of nondiabetic control (NC) rats that were given normal saline, group 2 included diabetic control (DC) rats, group 3 had diabetic rats treated with metformin (DM) (the initial dose was 300 mg/kg bw/day given by oral gavage for 2 weeks which was then increased to 500 mg/kg bw/day for 4 weeks), group 4 included diabetic rats that were given olive oil (DO) (1 mL/100 g bw/day), group 5 had diabetic rats treated with olive oil and metformin (DOM) (diabetic rats were given olive oil (1 mL/100 g bw/day) orally for six weeks, and then the rats were treated daily with metformin (300 mg/kg bw/day) by oral gavage for two weeks followed by increasing the dose of metformin to 500 mg/kg bw/day for four weeks), and group 6 included diabetic rats that were given olive oil pretreatment (DOP) (1 mL/100 g bw/day) for 2 weeks before the induction of diabetes.

#### 2.3.2. Oral Glucose Tolerance Test

Prior to overnight fasting and administration of D-glucose, blood was collected from the tail tip after 0, 30, 60, 90, and 120 minutes of administration.

### 2.4. Biochemical Analysis

Blood for biochemical analysis was collected via intraorbital sinus, and serum preparation was done by centrifugation (Sigma-Aldrich, USA) at 4000 rpm for 10 minutes. The serum was kept at −80°C for biochemical analysis.

#### 2.4.1. Lipid Profile

Cholesterol, high-density lipoprotein (HDL), low-density lipoprotein (LDL), and triacylglycerol (TAG) were measured in serum using CHOL Flex® Reagent Cartridge, LDL Flex® Reagent Cartridge, and TRIG Flex® Reagent Cartridge (Siemens Healthcare Diagnostics Inc., Newark, USA), respectively, and measurements were taken according to the manufacturer's instructions.

#### 2.4.2. Determination of Liver Function

Alkaline phosphatase (ALP), aspartate aminotransferase (AST), alanine aminotransferase (ALT), albumin (ALB), bilirubin (BIL), and total protein (TP) were measured using ALP Flex® Reagent Cartridge, AST Flex® Reagent Cartridge, ALTI Flex® Reagent Cartridge, ALB Flex® Reagent Cartridge, BIL Flex® Reagent Cartridge, and TP Flex® Reagent Cartridge (Siemens Healthcare Diagnostics Inc., Newark, USA), respectively, and experiments were performed according to the manufacturer's instructions.

#### 2.4.3. Determination of Kidney Function

Creatinine (CREA), uric acid (URCA), and blood urea nitrogen (BUN) were measured using CREA Flex® Reagent Cartridge, URCA Flex® Reagent Cartridge, and BUN Flex® Reagent Cartridge (Siemens Healthcare Diagnostics Inc., Newark, USA), respectively. All experiments were performed according to the manufacturer's instructions.

### 2.5. Histopathological Examination

The liver, kidney, and pancreas from all the six groups of rats were fixed with 10% buffered formalin for 24 hours. Tissues were embedded in paraffin blocks and sectioned at approximately 3-5 *μ*m thickness and were then stained with hematoxylin and eosin (H&E). Stained sections were examined for histological changes using light microscopy (Olympus BX51TF), and representative images were captured with an Olympus DP 72 camera.

### 2.6. Statistical Analysis

All the data were analyzed using SPSS (Statistical Package for the Social Sciences) version 20.0 software (SPSS Inc., Chicago, IL, USA). The data were presented as mean ± standard deviation (SD). Significance among different groups was determined by using the one-way analysis of variance (ANOVA) test. A *P* value of less than 0.05 was taken as having a statistically significant difference.

## 3. Results

### 3.1. Effect of Olive Oil on the Biochemical Parameters of STZ-Induced Diabetic Rats

#### 3.1.1. Effect on the Lipid Profile

Nonsignificant changes were observed in the serum lipid profile of DC rats when compared to NC rats. The serum level of LDL in DO and DOP groups was significantly higher than that in the NC group (*P* = 0.025 and *P* = 0.001, respectively), but there was no significant difference in DM and DOM groups. The TAG serum level was significantly higher in the DM group than in NC and DC groups (*P* = 0.0001 and *P* = 0.0001, respectively), while it was higher in the DOM group than in the NC group (*P* = 0.009). TAG levels were not significantly different in DO and DOP groups when compared to NC and DC groups. No significant differences in the CHO and HDL serum level were observed among all the treated groups when compared to NC and DC groups (Table 1).

#### 3.1.2. Effect on Liver Function

There was a nonsignificant difference in the serum AST, TP, BIL, and ALB of DC rats when compared to NC rats. The AST serum level in the DM group was significantly higher than that in NC and DC groups (*P* = 0.001 and *P* = 0.029, respectively). The AST level was also significantly higher in the DOM group than in the NC group (*P* = 0.049); however, there was no remarkable difference in DO and DOP groups when compared to NC and DC groups. On the other hand, the ALT serum level in DC, DM, DO, DOM, and DOP groups was noticeably more than that in the NC group (*P* = 0.0001, *P* = 0.0001, *P* = 0.0001, *P* = 0.0001, and *P* = 0.022, respectively) but was considerably lower in the DOP group than in the DC group (*P* = 0.0001). The ALP serum level in DC, DM, DO, DOM, and DOP groups was notably more than that in the NC group (*P* = 0.0001, *P* = 0.0001, *P* = 0.0001, *P* = 0.0001, and *P* = 0.023, respectively). It was also significantly higher in the DO group but lower in the DOP group than in the DC group (*P* = 0.0001 and *P* = 0.015, respectively). No meaningful differences in BIL serum level were observed among all the treated groups when compared to NC and DC groups. But the TP serum level was remarkably higher in the DO group than in NC and DC groups (*P* = 0.017 and *P* = 0.004, respectively) but was appreciably higher in the DOP group than in the DC group (*P* = 0.020); also, the changes were nonsignificant for the DOM group. The ALB serum level was significantly higher in DO and DOM groups than in the DC group (*P* = 0.019 and *P* = 0.004, respectively) but was not significantly different in DM and DOP groups when compared to NC and DC groups (Table 2).

#### 3.1.3. Effect on Kidney Function

There was a nonsignificant difference in the serum BUN, CREA, and URCA of DC rats when compared to NC rats. BUN and URCA serum levels in the DM group were significantly higher (*P* = 0.041 and *P* = 0.006, respectively) but were not significantly different in DO, DOM, and DOP groups in comparison to NC and DC groups. No significant differences in CREA serum level were observed among all the treated groups when compared to NC and DC groups (Table 3).

### 3.2. Effect of Olive Oil on the Histopathology of the Pancreas, Liver, and Kidney of STZ-Induced Diabetic Rats

#### 3.2.1. Effect on the Histopathology of the Endocrine Pancreas (Islets of Langerhans)

**Figure 1:**
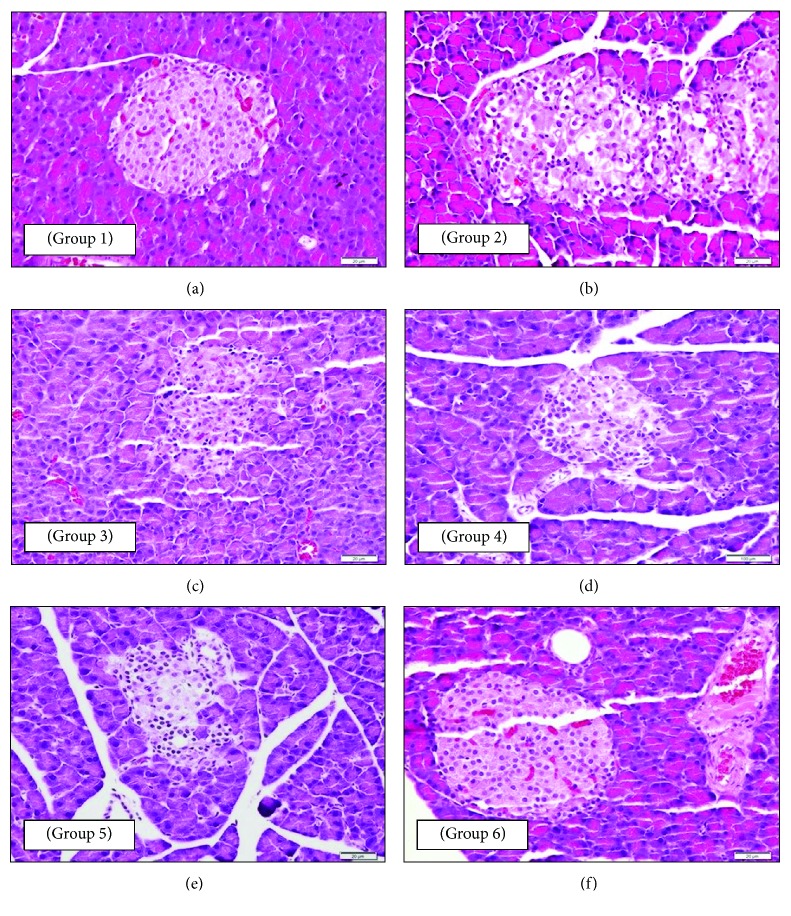
Photomicrographs of a section of the endocrine pancreatic tissue of rats: (a—group 1: NC) normal pancreatic structure and exocrine acini surrounding the islets of Langerhans (400x magnification); (b—group 2: DC) disorganized islets of Langerhans and clusters of inflammatory cells (*β*-cells show an unstained vacuolated cytoplasm and dark-stained degenerated nuclei) (400x magnification); (c—group 3: DM) normal islets of Langerhans and blood sinusoids (100x magnification); (d—group 4: DO) islets of Langerhans with a normal-looking population of *β*-cells (100x magnification); (e—group 5: DOM) normal islets of Langerhans with a normal population of *β*-cells and absence of any degenerative change (400x magnification); (f—group 6: DOP) normal islets of Langerhans with a normal *β*-cell population similar to that of group 1 (NC) indicating protection from the damaging effect of STZ (400x magnification). All sections were stained with H and E stain and viewed with a light microscope.

Histopathological examination of pancreatic tissue revealed that in untreated nondiabetic control (NC) rats, the islets of Langerhans as well as circumscribed masses surrounded by deeply stained pancreatic exocrine acini appeared normal (Figure 1(a)). *β*-cells could be identified partially with their location and their smaller dark nuclei, relative to those of *α*-cells. The cell clusters were separated by thin-wall blood sinusoids. In the pancreas of untreated diabetic control (DC) rats, islets of Langerhans appeared disorganized. *β*-cells showed an unstained vacuolated cytoplasm and dark-stained degenerated nuclei. Clusters of inflammatory cells could also be seen among the cells (Figure 1(b)). In contrast, the DM group showed that islets of Langerhans have a more or less normal cell population with the absence of degenerative changes as observed in the DC group. Blood sinusoids also appeared to be normal (Figure 1(c)). Light microscopy examination of the pancreatic tissue of the DO group revealed that islets of Langerhans have a normal-looking population of *β*-cells (Figure 1(d)). The pancreatic tissue of the DOM group showed normal islets of Langerhans with a normal population of *β*-cells and absence of any degenerative changes observed in the DC group (Figure 1(e)). In the DOP group, islets of Langerhans appeared normal with a normal *β*-cell population, much like the NC group indicating protection from the damaging effect of STZ (Figure 1(f)).

#### 3.2.2. Effect on the Histopathology of the Liver

**Figure 2:**
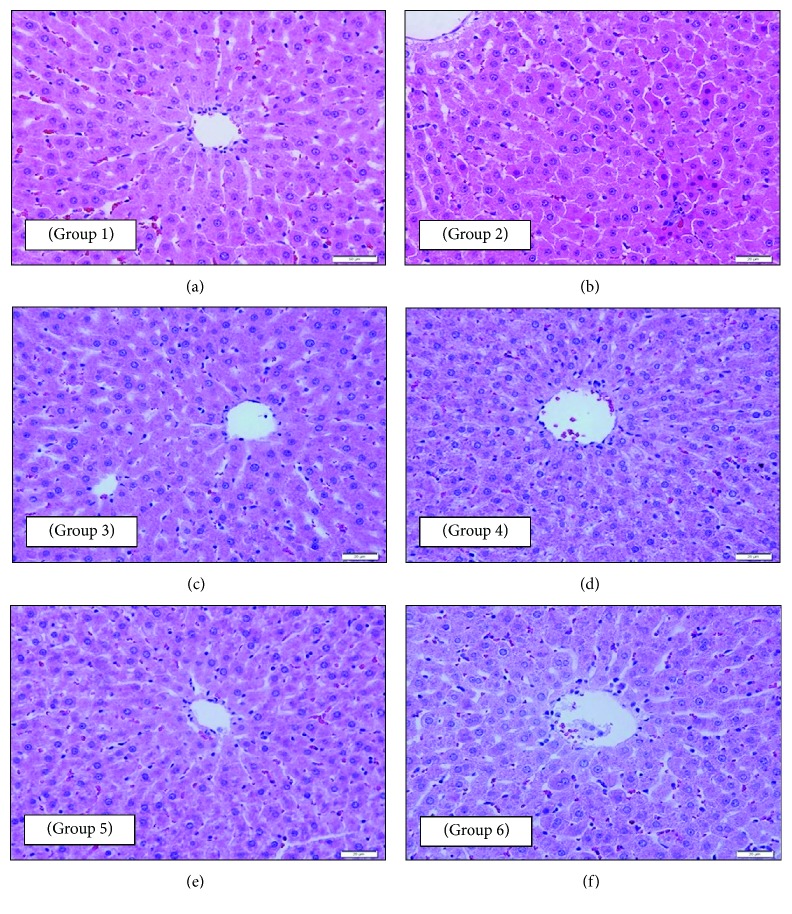
Photomicrographs of a section of the liver tissue of rats: (a—group 1: NC) almost normal hepatic structure (hepatocytes being radially arranged around the central vein and presence of von Kupffer cells lining the sinusoidal spaces) (200x magnification); (b—group 2: DC) numerous apoptotic dark-stained shrunken hepatocytes with small degenerated nuclei (400x magnification); (c—group 3: DM) nearly normal radially arranged hepatocytes around the central vein (it also shows that blood sinusoidal spaces and their von Kupffer cells are like those of the NC group) (400x magnification); (d—group 4: DO) almost normal hepatic structure with radially arranged hepatocytes around the central vein with the absence of any degenerated apoptotic cells (it also shows that hepatic sinusoidal spaces and von Kupffer cells are like those of the NC group) (400x magnification); (e—group 5: DOM) almost normal hepatic structure with radially arranged hepatocytes around the central vein (it also shows the presence of von Kupffer cells lining the sinusoidal spaces) (400x magnification); (f—group 6: DOP) almost normal hepatic structure with radially arranged hepatocytes around the central vein (it also shows active and healthy hepatocytes with active vesicular nuclei like that of the NC group) (400x magnification). All sections were stained with H and E stain and viewed with a light microscope.

Observation of the hepatic tissue of the untreated nondiabetic control (NC) rats showed histological features like radially arranged hepatocytes around the central vein. The cells have an acidophilic cytoplasm and rounded central vesicular euchromatic nuclei with well-defined nucleoli. The hepatocyte plates are separated by thin-walled blood sinusoids lined by flat endothelial cells. Occasionally, the prominent nuclei of von Kupffer cells were observed (Figure 2(a)). The section of the hepatic tissue of untreated diabetic control (DC) rats showed an increase in apoptotic hepatocytes (shrunken and dark-stained cells with small degenerated nuclei) (Figure 2(b)). The liver tissue sectioned from the DM rats showed nearly normal radially arranged hepatocytes around the central vein. Blood sinusoidal spaces and their von Kupffer cells are similar to those of the NC group (Figure 2(c)). The liver tissue taken from the DO rats exhibited almost normal hepatic structure with radially arranged hepatocytes around the central vein with the absence of any degenerated apoptotic cells. The hepatic sinusoidal spaces and von Kupffer cells are similar to those of the NC group (Figure 2(d)). Interestingly, the hepatic tissue of the DOM rat revealed relatively normal hepatic structure with radially arranged hepatocytes around the central vein. von Kupffer cells lining sinusoidal spaces were evident (Figure 2(e)). Meanwhile, the liver tissue of the DOP rat showed almost normal radially arranged hepatocytes around the central vein. Hepatocytes looked active and healthy with active vesicular nuclei similar to those observed in the NC group. The normal appearance of sinusoidal spaces and their lining by von Kupffer cells were exhibited (Figure 2(f)).

#### 3.2.3. Effect on the Histopathology of the Kidney

**Figure 3:**
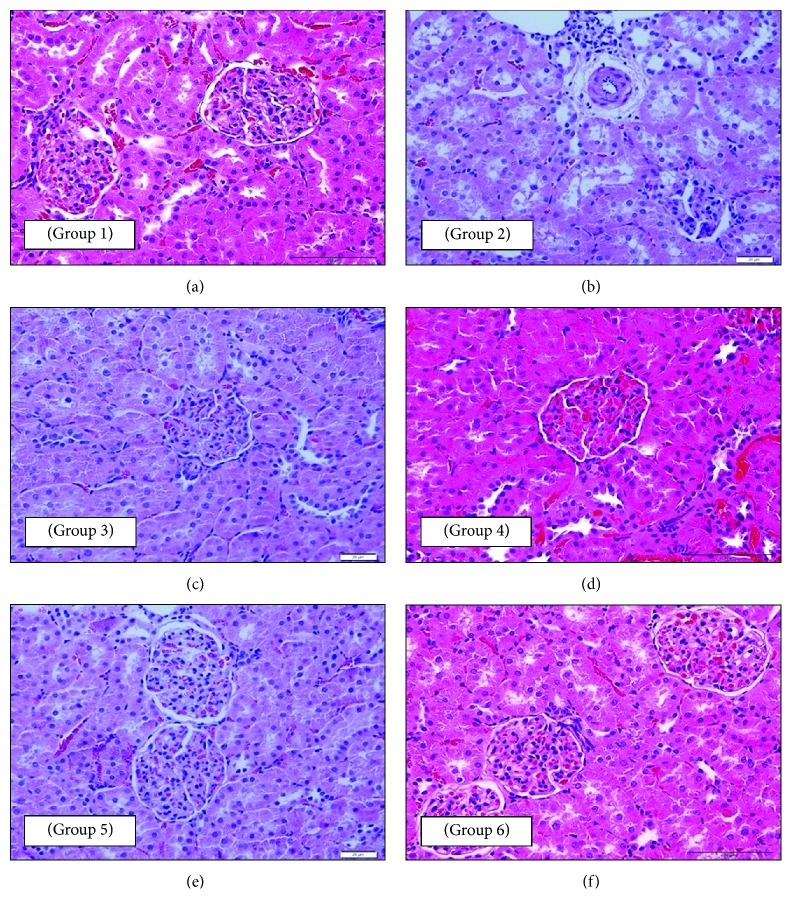
Photomicrographs of a section of the kidney tissue of rats: (a—group 1: NC) normal renal corpuscles with normal glomerular capillaries and renal tubules (40x magnification); (b—group 2: DC) deformity of renal corpuscle with atrophy of glomerular capillaries and unstained degenerated cytoplasmic regions in the renal tubular epithelium and arterioles showing diffused muscular media thickening with perivascular edema, fibrosis, and minimal interstitium lymphocytic infiltrate (400x magnification); (c—group 3: DM) normal renal corpuscles and glomeruli (it also shows normal renal tubules) (400x magnification); (d—group 4: DO) marked protection from diabetic changes with renal corpuscle, glomeruli, and tubules looking like those of the NC group (200x magnification); (e—group 5: DOM) marked protection against diabetic changes (it also shows normal and healthy renal corpuscles, glomeruli, and tubules) (400x magnification); (f—group 6: DOP) marked protection against diabetic changes (it also shows normal and healthy renal corpuscles, glomeruli, and tubules like those of the NC group) (200x magnification). All sections were stained with H and E stain and viewed with a light microscope.

Renal histopathology showed that nondiabetic control (NC) rats have a normal cortex and medulla (Figure 3(a)). Renal corpuscles are the main histological feature of the cortex. The outer layer of Bowman's capsule lined by the simple squamous epithelium was clearly shown. The glomerulus looked as clusters of capillaries covered by the inner layer of Bowman's capsule. Renal tubules in the cortex include mainly the proximal and distal parts of the nephron. These are lined by the cuboidal epithelium. They differ from each other in the height of the cells and width of the lumen. Capillaries were seen between tubules. The section from the untreated diabetic control (DC) rat kidney tissue showed deformed renal corpuscles with atrophy of glomerular capillaries. Renal tubules showed that the lining cells exhibited unstained degenerated cytoplasmic regions. Arterioles showing diffused muscular media thickening with perivascular edema, fibrosis, and minimal interstitium lymphocytic infiltrate were shown (Figure 3(b)). The kidney tissue of the DM rats showed that normal renal corpuscles and glomeruli were dominating (Figure 3(c)). Renal tubules also looked normal. Sections of the kidney from DO rats showed marked protection from diabetic changes (Figure 3(d)). Renal corpuscles, glomeruli, and tubules looked similar to those seen in the NC group. The renal tissue of the DOM group revealed marked protection against diabetic changes (Figure 3(e)). Renal corpuscles, glomeruli, and tubules looked normal and healthy like those of the NC group. The renal tissue of DOP rats showed marked protection against diabetic changes (Figure 3(f)).

## 4. Discussion

The protective effect of virgin olive oil was investigated in this study by observing the histopathological changes and biochemical parameters against STZ-induced diabetic rats. An abnormality in the lipid profile is one of the commonest complications associated with diabetes mellitus [16]. In the present study, it has been observed that there were nonsignificant differences in the serum lipid profile of DC rats when compared to NC rats. Also, no significant differences in CHO and HDL serum levels were observed among all the treated groups when compared to NC and DC groups. This could be attributed to the short experimental period. In this study, TAG in diabetic rats treated with metformin (DM) in combination with olive oil (DOM) was significantly increased after 6 weeks when compared with that in the NC group. Also, LDL in diabetic rats treated with olive oil (DO) and in diabetic rats pretreated with olive oil (DOP) was significantly increased after 6 weeks when compared with that in the NC group. Our findings are consistent with the study where the absence of lipid abnormalities in DC rats was found [17]. In accordance with our results, a significant increase was observed in TAG level in DM rats in another study when compared to the NC group [18]. However, other researchers have reported significant changes in lipid abnormalities [19].

The liver function test revealed no significant variation in the serum level of AST in the DC group compared to the NC group. In contrast, the serum level of ALT in DC and all the treated groups was significantly higher (*P* = 0.0001) than that in the NC group. The serum level of ALT decreased significantly (*P* = 0.0001) in the DOP group when compared to the DC group. The serum level of AST was significantly increased (*P* = 0.001 and *P* = 0.029, respectively) in the DM group when compared to NC and DC groups and in the DOM (*P* = 0.049) group when compared to the NC group.

Moreover, in the present study, some biochemical tests reflecting hepatocyte function, such as serum BIL, ALB, TP, and ALP, were performed. ALP is a potent anti-inflammatory mediator that can protect tissues from damage resulting from injury. Elevated activity of ALP (*P* = 0.0001) was observed in the DC group of rats when compared to the NC group. This observation of our study is supported by a previous study which reported increased ALP activity in experimentally diabetic rats [20]. The increased activity of this enzyme in serum may be a result of diabetes-induced damage to the tissues. In the present study, it has been shown that the serum level of ALP remained elevated in DM and DOM groups (*P* = 0.0001 and *P* = 0.0001, respectively) when compared to the NC group and in the DO group (*P* = 0.0001 and *P* = 0.0001, respectively) when compared to NC and DC groups. In contrast, the serum level of ALP in the DOP group was significantly decreased (*P* = 0.015) when compared to the DC group and significantly increased (*P* = 0.023) when compared to the NC group. Moreover, no significant variation has been observed in the serum BIL, TP, and ALB levels between the NC group and the DC group. The serum level of BIL among all groups was within normal range. Treatment of diabetic rats with olive oil (DO) caused a noticeable elevation in the serum TP level (*P* = 0.017 and *P* = 0.004, respectively) as compared to NC and DC groups. The serum level of ALB was found to be significantly increased in DO and DOM groups (*P* = 0.019 and *P* = 0.004, respectively) when compared to the DC group. These results agree with the study which reported that treatment of rats with STZ alone did not cause any significant changes in levels of TP, AST, and ALT [21].

The serum BUN, CREA, and URCA levels are the markers of nephrotoxicity implicated in the diagnosis of kidney damage [22]. In the present study, no significant variation in the serum BUN, CREA, and URCA levels between the NC and the DC rats as well as the treated rats was observed. This could be attributed to the species of rats being studied or the short experimental period. The results of this study showed that there is no significant increase in the level of serum CREA in all the treated groups when compared to the NC group. In contrast, the level of serum BUN in the DM group was significantly higher (*P* = 0.008 and *P* = 0.041, respectively) when compared to NC and DC groups. However, CREA is a more reliable indicator of renal function than BUN because BUN is not the most sensitive indicator of renal damage and is more likely to be affected by dietary and physiologic conditions that are not related to renal function [23].

Our findings are consistent with the study of Menon et al. where it was found that there was no significant increase in serum CREA and URCA levels in the DC group when compared to the NC group [23]. However, serum BUN levels in the DC group were found to be significantly increased when compared to those in the NC group. However, the observed significant increase in BUN in the DC and treated diabetic groups may be unrelated to renal function. However, contrary to our results, some researchers have observed that there was a significant increase in serum concentrations of BUN, UACA, and CREA in the DC group when compared to the NC group [24].

In this study, the histopathological examination of the sections of the pancreas, liver, and kidney from the DC group of rats showed massive pathological changes as compared to the normal structure observed in the NC group. The pancreatic tissue showed disorganization of the endocrine islets of Langerhans with *β*-cells showing an unstained vacuolated cytoplasm and dark-stained degenerated nuclei. Clusters of inflammatory cells could also be seen among the cells. The histopathological changes observed in the DC group were like those reported previously [25]. In contrast, the pancreatic tissue of the DM group showed a more or less normal population of islets of Langerhans, while the absence of degenerative changes was observed in the DC group. Further, this result agrees with the study where the same results were reported [26]. The pancreatic tissue of the DO group showed islets of Langerhans with a normal-looking population of *β*-cells. This finding is in agreement with the report of Al-Janabi et al. which reported a more healthy architecture of islets of Langerhans and acinar cells after the treatment with olive leaf extracts [26]. Treatment with metformin (DM) in combination with olive oil (DOM) showed an improvement in islets of Langerhans and normal population of *β*-cells, and the absence of any degenerative changes was observed in the DC group. These findings suggest that olive oil potentiates the effect of metformin to protect diabetic rat pancreas against oxidative stress and damage. Meanwhile, the pancreatic sections from the DOP group showed normal islets of Langerhans with a normal *β*-cell population that was much similar to that of the NC group indicating that olive oil exerted a protective effect against the destructive effect of STZ. Therefore, this protective effect may be attributed to the prophylactic intake of olive oil.

Vital organ examination, especially of the liver and kidney, showed that they are target organs for diabetic complication. Histopathological examination of the liver from the DC group showed pathological changes indicating degenerative change such as the presence of numerous dark-stained shrunken hepatocytes and small dark nuclei (apoptosis). Treatment with metformin (DM), olive oil (DO), and a combination of olive oil and metformin (DOM), respectively, restored the normal structural organization of the liver tissue in STZ diabetic rats, thereby exhibiting a protective role against hepatic damage. These histopathological changes observed in the DO group were similar to those reported previously [27]. Pretreatment with olive oil (DOP) was found to preserve the normal structure of the rat liver indicating a preventive or prophylactic role of olive oil.

In the present study, the kidney tissue of the DC group showed deformity of renal corpuscles with atrophy of glomerular capillaries. Additionally, renal tubules showed unstained degenerated cytoplasmic regions of the lining epithelium, while arterioles showed diffuse muscular media thickening with perivascular edema, fibrosis, and minimal interstitium lymphocytic infiltrate. These results are well supported by the study of Gopal et al. (2013) which showed severe destruction as well as glomerular sclerosis in the kidney of diabetic rats [28]. Furthermore, the DOP group in the present study showed protection against diabetic changes. Renal corpuscles, glomeruli, and tubules looked healthy like those in the NC group. These findings indicate the beneficial use of olive oil as a protective measure to decrease the risk of developing diabetes.

The histopathological data from this study strongly implies that the combination of olive oil with oral hypoglycemic agents may be a valuable adjuvant therapy to achieve and/or maintain glycemic control and possibly reduce or delay the onset of diabetic complications. We recommend further investigations on humans to study the complementary effect of the combination of olive oil and metformin on other body tissues.

### 4.1. Significance of the Study

This study shows the ameliorative effect of olive oil on diabetic rats that can be beneficial to maintain a glycemic control in diabetes and prevent the onset of diabetic complications. This study will further help in the research to uncover the critical area of how to achieve the management of diabetes and prevention of secondary diseases arising out of hyperglycemia. Thus, a new theory of pretreatment with olive oil as an adjuvant and a prophylactic supplement against diabetes and in antioxidant therapy in combination with hypoglycemic drugs in the management of diabetes mellitus may be arrived at.

## Figures and Tables

**Table 1:** Lipid profiles of different groups of rats during the experiment and after the induction of diabetes by streptozotocin. Data was expressed as mean ± standard deviation (SD). Significances among different groups were determined by using the one-way analysis of variance (ANOVA) test. ^1^**P**: significance versus control, ^2^**P**: significance versus diabetic control.

Rat groups	Cholesterol (mmol/L)	High-density lipoprotein (mmol/L)	Low-density lipoprotein (mmol/L)	Triacylglycerol (mmol/L)
Group 1: nondiabetic control (NC)	1.37 ± 0.13	1.30 ± 0.10	0.23 ± 0.02	0.63 ± 0.13
Group 2: diabetic control (DC)	1.39 ± 0.10 ^1^*P* = 0.899	1.11 ± 0.21 ^1^*P* = 0.127	0.33 ± 0.03 ^1^*P* = 0.097	0.82 ± 0.17 ^1^*P* = 0.540
Group 3: diabetic and metformin (DM)	1.47 ± 0.23 ^1^*P* = 0.486, ^2^*P* = 0.590	1.31 ± 0.19 ^1^*P* = 0.943, ^2^*P* = 0.111	0.30 ± 0.06 ^1^*P* = 0.196, ^2^*P* = 0.658	1.98 ± 0.65 ^1^**P** = 0.0001, ^2^**P** = 0.0001
Group 4: diabetic and olive oil (DO)	1.42 ± 0.42 ^1^*P* = 0.719, ^2^*P* = 0.828	1.31 ± 0.22 ^1^*P* = 0.954, ^2^*P* = 0.114	0.36 ± 0.04 ^1^**P** = 0.025, ^2^*P* = 0.599	1.15 ± 0.47 ^1^*P* = 0.077, ^2^*P* = 0.273
Group 5: diabetic, olive oil, and metformin (DOM)	1.36 ± 0.25 ^1^*P* = 0.955, ^2^*P* = 0.857	1.26 ± 0.09 ^1^*P* = 0.722, ^2^*P* = 0.230	0.31 ± 0.12 ^1^*P* = 0.144, ^2^*P* = 0.782	1.42 ± 0.44 ^1^**P** = 0.009, ^2^*P* = 0.052
Group 6: diabetic and olive oil pretreatment (DOP)	1.34 ± 0.29 ^1^*P* = 0.848, ^2^*P* = 0.757	1.20 ± 0.19 ^1^*P* = 0.387, ^2^*P* = 0.472	0.42 ± 0.22 ^1^**P** = 0.001, ^2^*P* = 0.126	1.04 ± 0.53 ^1^*P* = 0.164, ^2^*P* = 0.466

**Table 2:** Liver functions of different groups of rats during the experiment and after the induction of diabetes by streptozotocin. Data was expressed as mean ± standard deviation (SD). Significances among different groups were determined by using the one-way analysis of variance (ANOVA) test. ^1^**P**: significance versus control, ^2^**P**: significance versus diabetic control.

Rat groups	Aspartate aminotransferase (U/L)	Alanine aminotransferase (U/L)	Alkaline phosphatase (U/L)	Bilirubin (*μ*mol/L)	Total protein (g/L)	Albumin (g/L)
Group 1: nondiabetic control (NC)	114.01 ± 8.27	49.20 ± 11.84	134.20 ± 21.79	2.83 ± 0.41	70.00 ± 2.28	11.17 ± 1.72
Group 2: diabetic control (DC)	127.00 ± 18.83 ^1^*P* = 0.274	106.00 ± 14.25 ^1^**P** = 0.0001	329.25 ± 24.19 ^1^**P** = 0.0001	2.60 ± 0.89 ^1^*P* = 0.663	68.40 ± 2.79 ^1^*P* = 0.505	9.60 ± 1.95 ^1^*P* = 0.070
Group 3: diabetic and metformin (DM)	153.52 ± 27.24 ^1^**P** = 0.001, ^2^**P** = 0.029	92.60 ± 18.40 ^1^**P** = 0.0001, ^2^*P* = 0.105	302.00 ± 73.70 ^1^**P** = 0.0001, ^2^*P* = 0.506	3.50 ± 1.22 ^1^*P* = 0.197, ^2^*P* = 0.099	65.83 ± 3.87 ^1^*P* = 0.074, ^2^*P* = 0.287	10.50 ± 1.87 ^1^*P* = 0.411, ^2^*P* = 0.291
Group 4: diabetic and olive oil (DO)	134.00 ± 14.37 ^1^*P* = 0.081, ^2^*P* = 0.554	91.16 ± 13.05 ^1^**P** = 0.0001, ^2^*P* = 0.074	568.33 ± 110.32 ^1^**P** = 0.0001, ^2^**P** = 0.0001	3.33 ± 1.21 ^1^*P* = 0.331, ^2^*P* = 0.176	75.67 ± 4.89 ^1^**P** = 0.017, ^2^**P** = 0.004	11.67 ± 1.37 ^1^*P* = 0.537, ^2^**P** = 0.019
Group 5: diabetic, olive oil and metformin (DOM)	136.67 ± 34.05 ^1^**P** = 0.049, ^2^*P* = 0.414	96.00 ± 11.47 ^1^**P** = 0.0001, ^2^*P* = 0.223	348.68 ± 94.53 ^1^**P** = 0.0001, ^2^*P* = 0.635	2.83 ± 0.41 ^1^*P* = 1.000, ^2^*P* = 0.663	72.50 ± 2.17 ^1^*P* = 0.277, ^2^*P* = 0.092	12.17 ± 0.98 ^1^*P* = 0.220, ^2^**P** = 0.004
Group 6: diabetic and olive oil pretreatment (DOP)	114.60 ± 15.37 ^1^*P* = 0.958, ^2^*P* = 0.296	67.64 ± 12.10 ^1^**P** = 0.022, ^2^**P** = 0.0001	226.17 ± 53.05 ^1^**P** = 0.023, ^2^**P** = 0.015	3.00 ± 1.10 ^1^*P* = 0.744, ^2^*P* = 0.457	74.17 ± 3.76 ^1^*P* = 0.074, ^2^**P** = 0.020	11.00 ± 0.63 ^1^*P* = 0.836, ^2^*P* = 0.104

**Table 3:** Kidney functions of different groups of rats during the experiment and after the induction of diabetes by streptozotocin. Data was expressed as mean ± standard deviation (SD). Significances among different groups were determined by using the one-way analysis of variance (ANOVA) test. ^1^**P**: significance versus control, ^2^**P**: significance versus diabetic control.

Rat groups	Blood urea nitrogen (mmol/L)	Creatinine (*μ*mol/L)	Uric acid (*μ*mol/L)
Group 1: nondiabetic control (NC)	7.23 ± 0.79	42.83 ± 5.38	55.17 ± 7.83
Group 2: diabetic control (DC)	7.84 ± 1.79 ^1^*P* = 0.597	49.80 ± 8.90 ^1^*P* = 0.055	48.80 ± 4.09 ^1^*P* = 0.281
Group 3: diabetic and metformin (DM)	10.25 ± 1.73 ^1^**P** = 0.008, ^2^**P** = 0.041	47.67 ± 5.54 ^1^*P* = 0.159, ^2^*P* = 0.549	65.83 ± 11.84 ^1^*P* = 0.062, ^2^**P** = 0.006
Group 4: diabetic and olive oil (DO)	7.22 ± 2.14 ^1^*P* = 0.988, ^2^*P* = 0.587	48.67 ± 2.50 ^1^*P* = 0.091, ^2^*P* = 0.750	49.33 ± 15.32 ^1^*P* = 0.300, ^2^*P* = 0.927
Group 5: diabetic, olive oil, and metformin (DOM)	7.02 ± 1.48 ^1^*P* = 0.843, ^2^*P* = 0.474	48.17 ± 5.27 ^1^*P* = 0.121, ^2^*P* = 0.646	55.67 ± 7.79 ^1^*P* = 0.929, ^2^*P* = 0.245
Group 6: diabetic and olive oil pretreatment (DOP)	8.93 ± 3.43 ^1^*P* = 0.125, ^2^*P* = 0.343	45.83 ± 3.97 ^1^*P* = 0.378, ^2^*P* = 0.268	52.67 ± 7.39 ^1^*P* = 0.655, ^2^*P* = 0.510

## Data Availability

The data used to support the findings of this study are included within the article.
